# Genomic insights about the effect of sodium-glucose cotransporter 2 inhibitors: a systematic review

**DOI:** 10.3389/fgene.2025.1571032

**Published:** 2025-05-30

**Authors:** Pavitraa Saravana Kumar, Yogapriya Chidambaram, G. Shree Devi, Vettriselvi Venkatesan, Ramesh Sankaran, Nagendra Boopathy Senguttuvan, Thanikachalam Sadagopan, Dorairaj Prabhakaran

**Affiliations:** ^1^ Department of Clinical Research, Sri Ramachandra Institute of Higher Education and Research (SRIHER), Chennai, Tamil Nadu, India; ^2^ Department of Emergency Medicine, Sri Ramachandra Institute of Higher Education and Research (SRIHER), Chennai, Tamil Nadu, India; ^3^ Department of Human Genetics, Sri Ramachandra Institute of Higher Education and Research (SRIHER), Chennai, Tamil Nadu, India; ^4^ Department of Cardiology, Sri Ramachandra Institute of Higher Education and Research (SRIHER), Chennai, Tamil Nadu, India; ^5^ Executive Director, Centre for Chronic Disease Control (CCDC), Delhi, India

**Keywords:** sodium-glucose cotransporter 2 inhibitors, heart failure, genomics, gene expression, inflammatory biomarker

## Abstract

**Introduction:**

Heart failure (HF) is a complex clinical syndrome with high morbidity and mortality, significantly burdening healthcare systems worldwide. Despite advances in therapy, effective treatment options remain limited. Sodium-glucose cotransporter 2 (SGLT2) inhibitors, initially developed for diabetes management, have demonstrated cardiovascular benefits, including reductions in HF hospitalizations and mortality. This systematic review examines the genomic effects of SGLT2 inhibitors in HF patients, focusing on gene expression, inflammatory biomarkers, and potential personalized treatment pathways.

**Methods:**

A systematic literature search of various databases was conducted up to November 2024, following PRISMA guidelines. Studies were included if they explored the genomic or molecular impacts of SGLT2 inhibitors in HF. Data extraction and analysis focused on gene expression changes, circulating biomarkers, and potential genomic mechanisms.

**Results:**

Of the 258 identified studies, three met the inclusion criteria. Key findings include: a) SGLT2 inhibitors downregulate pro-inflammatory genes in adipose tissue, reducing immune cell infiltration and ferroptosis; b) Genetic evidence highlights CXCL10 as a mediator of anti-inflammatory effects, with its inhibition linked to reduced HF risk; c) LRRTM2, a protein associated with synaptic formation, emerged as a critical mediator, with genetic links to reduced HF risk via SGLT2 inhibition.

**Discussion:**

This review underscores the genomic mechanisms through which SGLT2 inhibitors provide cardiovascular benefits. Key insights into gene expression modulation and protein interactions reveal pathways for personalized HF treatment. While findings are promising, further large-scale studies are needed to validate these mechanisms and their clinical implications.

**Systematic Review Registration:**

https://www.crd.york.ac.uk/prospero/, identifier CRD42024614674.

## 1 Introduction

Heart failure (HF) is a multifaceted clinical syndrome characterized by symptoms such as dyspnea, exercise intolerance, fatigue, palpitations, and pedal edema, arising from the heart’s inability to meet the body’s demands. This inability could be due to impaired ventricular filling, impaired pumping, or a combination of these. An estimated 23 million individuals worldwide suffer from heart failure, of which 50% have HF with reduced ejection fraction (HFrEF) (Murphy et al., 2020).

In India, heart failure is the leading cause of hospitalization among patients with cardiac conditions, affecting approximately 1% of the general population annually, equating to 8–10 million individuals. However, among those aged 65–79 years, the prevalence differs significantly, with 5%–10% of hospitalizations attributed to heart failure. Heart failure is associated with significant morbidity and mortality. In patients with chronic heart failure, the 1-year mortality rate is 7.2%, and the 1-year hospitalization rate is 31.9%. These rates rise to 17.4% and 43.9%, respectively in patients hospitalized for acute heart failure (Maggioni et al., 2013). Hospitalization rates are even higher (10%–20%) among those over 80 (National Heart Failure Registry, 2024) Notably, the 5-year mortality rate may go up to 45%–60% (Kim et al., 2022).

Based on the ejection fraction (EF) of the left ventricle, the patients are divided into three groups: reduced EF (EF < 40%; HFrEF); mid-range EF (EF between 40% and 49%; HFmrEF); and preserved EF (EF > or = 50%; HFpEF) (McDonagh et al., 2021). Patients with heart failure continue to have extremely poor prognoses and quality of life despite advancements in treatment. Therefore, creating novel treatments and methods to stop and treat heart failure is essential (Muscoli et al., 2022). Until recently, no new drug reduced clinical events in patients with heart failure since the inception of angiotensin-converting enzyme (ACE) inhibition/angiotensin receptor blockers (ARB), beta-blockers, and aldosterone inhibitors. Angiotensin receptor blockers with neprilysin inhibitors (ARNI) and sodium-glucose transport 2 inhibitors (SGLT2 inhibitors) are the new addition to the armamentarium of heart failure drugs, making heart failure therapy’s four pillars (Ndiaye et al., 2023).

**FIGURE 1:**
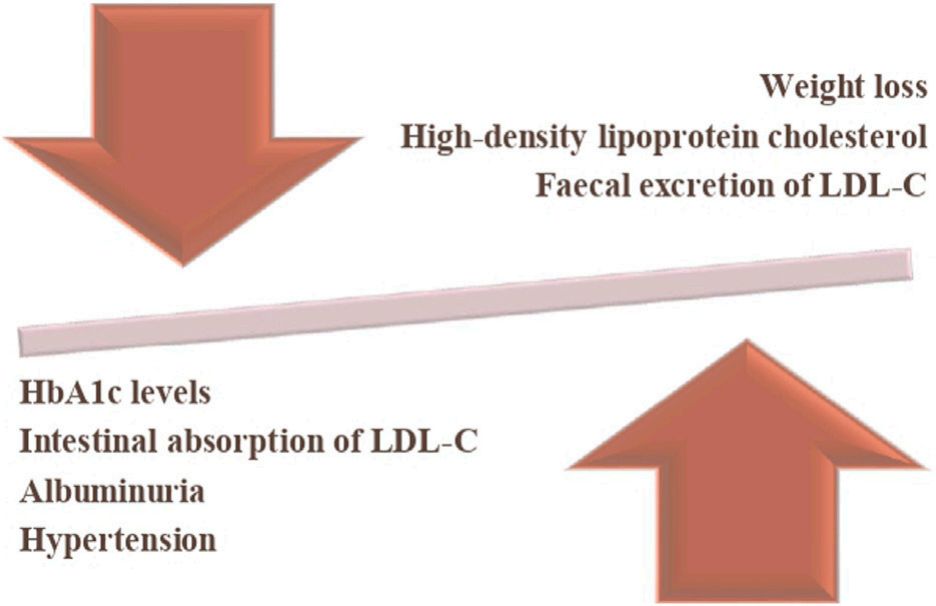
Benefits of SGLT2 inhibitors. The downward arrow represents a reduction in HbA1c levels, intestinal absorption of LDL cholesterol (LDL-C), albuminuria, and hypertension. The upward arrow indicates an increase in high-density lipoprotein cholesterol (HDL-C), and faecal excretion of LDL-C and weight reduction. The balance symbolizes the overall impact of the intervention, demonstrating its potential benefits in metabolic regulation.

SGLT2 inhibitors have shown great promise as a class of drugs with pleiotropic effects that go beyond the glycaemic management for which they were originally introduced. SGLT2 inhibitors have demonstrated significant reductions in cardiovascular mortality, heart failure hospitalizations, major adverse cardiovascular events, and potentially other cardiovascular outcomes (Molina and Valdez, 2024). Some of the benefits are presented in Figure 1.

Regardless of insulin secretion or action, SGLT2 inhibitors work by preventing renal glucose reabsorption, which increases glycosuria and lowers plasma glucose levels (Bailey et al., 2022). Inhibitors of the sodium-glucose co-transporter protein 2 (SGLT2) were first developed to treat type 2 diabetes. They are a class of oral antihyperglycemic drugs that are insulin-independent (Simes and MacGregor, 2019). Recent studies have undoubtedly proven their role as a potential cardio-reno-metabolic modifier that culminates in better clinical outcomes for patients with heart failure (Neuen et al., 2022). Building on these insights, genetic research has begun to unravel how individual variations in genes related to glucose transport metabolism and renal function may influence patient responses to SGLT2 inhibitors.

Few genomic studies have recently shed light on the genetic factors influencing the response to SGLT2 inhibitors, highlighting potential genetic biomarkers and pharmacogenomic profiles linked to their efficacy (Guo et al., 2024; Luo et al., 2024). There could be potentially other mechanisms that need exploration for which whole genome sequencing is required. While some animal studies are available, future research should focus on gene expression and other genetic aspects of SGLT2 inhibitors (Wu et al., 2023; Chen et al., 2022). These advancements may pave the way for personalized treatment approaches, enhancing therapeutic outcomes for heart failure patients and unlocking new possibilities for treatment. Despite this promising potential, few studies have specifically examined the impact of SGLT2 inhibitors on genetic variations in heart failure, and their findings remain inconclusive. To address this knowledge gap, we conducted a systematic review to explore the genetic effects of SGLT2 inhibitors in the context of heart failure.

## 2 Methodology

This systematic review was conducted and reported following the Preferred Reporting Items for Systematic Reviews and Meta-Analyses (PRISMA) guidelines. It has been submitted to and registered with PROSPERO (Registration Number: CRD42024614674).

### 2.1 Search strategy and sources

To find pertinent studies, a comprehensive and systematic literature search was carried out in multiple databases, including PubMed, Cochrane Library, Embase, and ClinicalTrials.gov, covering all studies published from inception to November 2024. The primary objective of this search was to identify relevant studies examining the effects of SGLT2 inhibitors on gene expression in patients with heart failure. A structured keyword search was developed using Medical Subject Headings (MeSH) terms and free-text words to maximize the specificity. A detailed list of keywords and search terms, such as “SGLT2 inhibitors,’’ “Sodium-glucose cotransporter-2 inhibitors,” “canagliflozin,” “dapagliflozin,” “empagliflozin,” “heart failure,” “genetics,” “genes,” “genomics,” “gene variations,’’ and “gene expression” was incorporated into the search strategy. To cover every facet of the topic and systematically to refine search results these terms were combined in a variety of ways using Boolean operators (AND, OR).

### 2.2 Eligibility criteria

The inclusion and exclusion criteria were as follows:

Inclusion:

• Studies focusing on human populations or preclinical models directly relevant to SGLT2 inhibitors.
• Must report on at least one pre-specified outcome (therapeutic efficacy, genetic/molecular impact, or safety).
• Subjects with heart failure (reduced and preserved ejection fraction).
• All types of study design.

Exclusion:

• Lack of clear outcome definitions or incomplete datasets.
• Articles other than English language.

### 2.3 Selection process

Two independent investigators (PSK and YC) performed the initial evaluation of the search results using predefined inclusion and exclusion criteria. After eliminating duplicates, the remaining studies were screened by their titles and abstracts to identify potentially relevant studies. The selected studies underwent full-text screening by two independent investigators (PSK and YC). The search was supplemented by reviewing the reference lists of the included studies. Any disagreement between the reviewers regarding study selection was resolved through discussion. If consensus could not be reached, a third and fourth senior reviewers (NBS and VV) were consulted for judgment. The senior reviewers independently assessed the study and offered a final decision, ensuring that we adhered to the inclusion and exclusion criteria. This approach ensured a thorough and unbiased selection process.

A thorough analysis of the full texts of potentially relevant articles was then conducted to ensure alignment with the research goals and questions. Articles that met the inclusion criteria and contributed to advancing knowledge on the cardiovascular benefits and gene expression of SGLT2 inhibitors were selected for the review.

### 2.4 Data extraction

Publication details (title, first author’s name, and publication year), exposure information (SGLT2 inhibitors, data source, and study population), outcome data (diseases or biomarkers, sample size, data source, and study population), and study designs were independently extracted by two authors (PSK and YC). NBS reviewed the extracted data to ensure its accuracy and completeness. A meta-analysis was planned if the included studies were sufficiently homogeneous in terms of data quantity and quality. However, due to the limited number of studies and their heterogeneity, we ultimately opted for a systematic review. Descriptive details of the studies are presented in Table 1.

**TABLE 1:** Descriptive data of original articles reviewed.

Year	Authors	Study design	Sample size	Outcome assessed	Conclusion
2024	Kasperova et al. (2024)	Cross-sectional clinical trial	N = 46, HFrEF subjects with SGLT2 inhibitors = 26, HFrEF subjects without SGLT2 inhibitors = 26	Gene expression analysis	The differential expression analysis revealed substantial downregulation, with significantly reduced transcript levels of IL6, IL1R1, IL1RAP, CCL2, CXCL2, and TNFAIP3 in subcutaneous adipose tissue (SAT) of patients treated with SGLT2 inhibitors
2024	Guo et al. (2024)	Two-sample, two-step MR approach	Data of HF obtained from the largest-scale GWAS: 47,309 HF cases and 930,014 controls	Inflammatory biomarkers - CXCL10 and LIF	This study presents genetic evidence highlighting the anti-inflammatory properties of SGLT2 inhibitors and their positive role in lowering the risk of heart failure. CXCL10 was identified as a potential mediator, introducing a novel pathway for heart failure treatment
2024	Luo et al. (2024)	Two-sample, two-step MR analysis	Data were obtained from the HERMES consortium: 47,309 HF cases and 930,014 controls	Plasma proteins - LRRTM2	LRRTM2 was identified among 4,907 plasma proteins, suggesting that SGLT2 inhibition may affect heart failure by modulating circulating LRRTM2 levels

Abbreviations: GWAS- Genome-wide association study; HERMES- heart failure molecular epidemiology for Therapeutic Targets; MR- mendelian randomization; N- sample size.

### 2.5 Risk of bias assessment

The risk of bias evaluation was conducted using the ROBINS-I tool, which assesses the risk of bias in non-randomized studies of interventions. It presents the risk of bias across seven domains for three studies: Kasperova et al. (2024), Guo et al. (2024), and Luo et al. (2024). Kasperova et al. (2024) is evaluated as having low risk of bias across all domains. In contrast, both Guo et al. (2024), Luo et al. (2024) show moderate risk of bias for confounding and serious risk of bias in the classification of interventions, but low risk of bias in the other five domains.

## 3 Results

**FIGURE 2:**
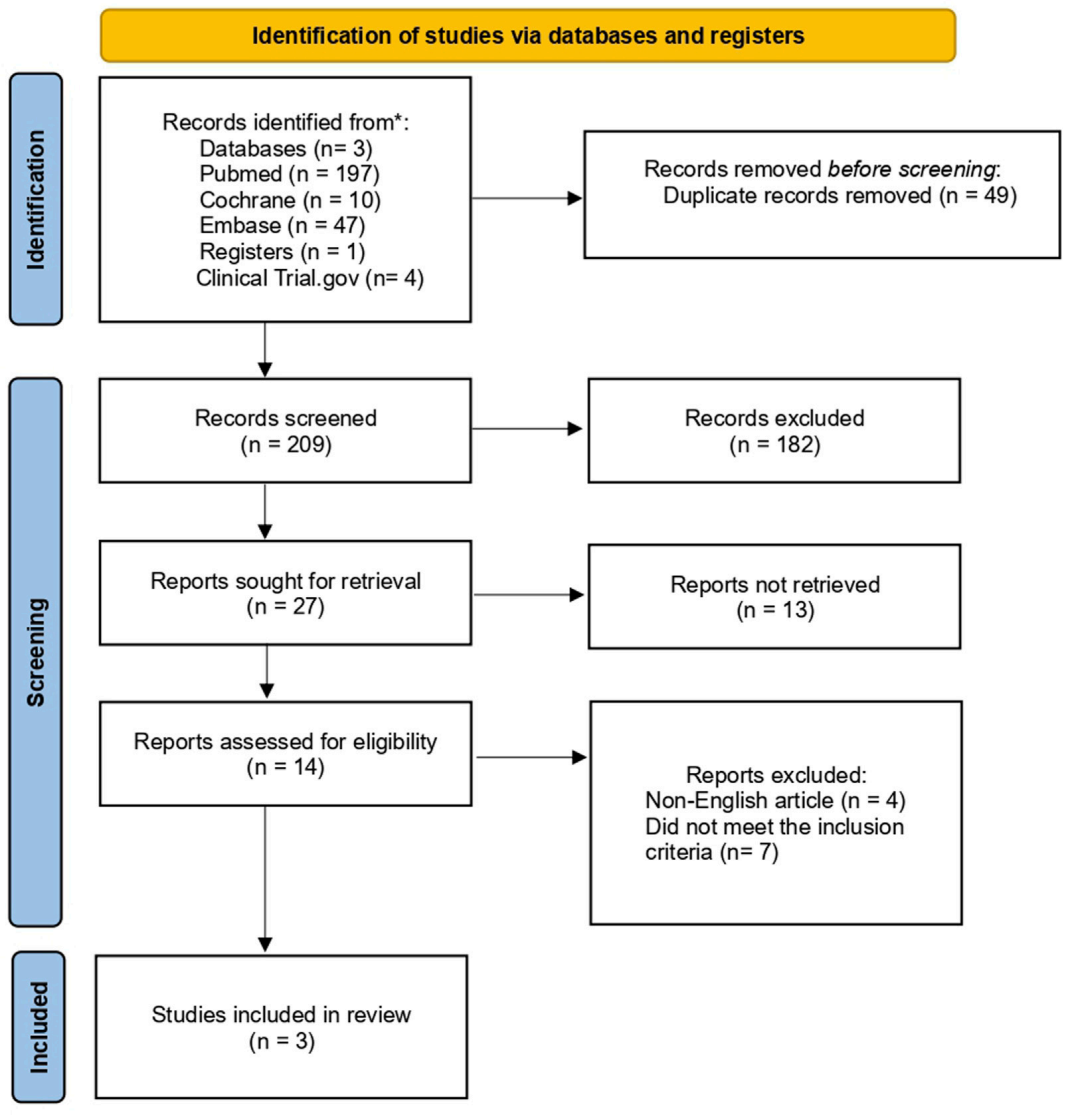
PRISMA flowchart.

A systematic search revealed 258 potentially eligible studies. Following the removal of 49 duplicates, titles and abstract screening were performed on the remaining studies, resulting in the exclusion of 182 articles due to a lack of genomic focus, non-heart failure population, and preclinical models without human validation. More studies were eliminated after a full-text review of the 27 that remained, due to insufficient genomic analysis, small sample sizes, poor methodology, and irrelevant genetic pathways. Only three studies ultimately satisfied all inclusion requirements, offering genomic information about how SGLT2 inhibitors affect heart failure (Figure 2).

### 3.1 Included studies

The included research was done in the Czech Republic (n = 1) and China (n = 2). One of them was a cross-sectional study and the other two used the Mendelian Randomization (MR) approach. These studies are heterogeneous in nature with respect to their design, studied genes, and their outcomes. Given the small number of publications, conducting a meta-analysis was not feasible. Therefore, we decided to report this solely as a systematic review.

### 3.2 Intervention results

Kasperova et al. (2024) conducted a cross-sectional study to assess the impact of SGLT2 inhibitors versus non-SGLT2 inhibitors on gene expression in individuals with severe heart failure. Group 1 (n = 26) received SGLT2 inhibitors for a mean duration of 258 ± 83 days, while Group 2 (n = 26) did not receive SGLT2 inhibitors. Gene expression analysis of epicardial adipose tissue (EAT) and subcutaneous adipose tissue (SAT) was performed using RNA sequencing, examining a total of 2,723 genes across both tissue types. The authors concluded that SGLT2 inhibitor treatment downregulates genes related to senescence, cell cycle arrest, and ferroptosis by shifting adipose tissue gene expression away from proinflammatory profiles, alongside reduced activity in inflammatory pathways like NF-κB and JAK/STAT. Concurrently, it enhances ether lipid production and activates tissue repair pathways such as TGFβ and WNT, potentially mitigating cardiovascular damage.

Adipose tissue inflammation and the emergence of metabolic disorders are significantly influenced by pro-inflammatory cytokines. Analysis of differential expression showed significant downregulation of transcripts, with markedly decreased levels of IL6 (Inflammatory Cytokine Interleukin-6), IL1R1 (Interleukin 1 receptor, type I), IL1RAP (interleukin 1 receptor accessory protein), CCL2 (chemokine (C-C motif) ligand 2), CXCL2 (Chemokine (C-X-C motif) ligand 2), and TNFAIP3 (Tumor Necrosis Factor Alpha-Induced Protein-3) in SAT among the patients who received SGLT2 inhibitors. A comparable reduction in gene expression was noted in EAT, though only TNFAIP3 achieved statistical significance (Kasperova et al., 2024).

Guo et al. (2024) used a two-sample Mendelian Randomization (MR) technique to examine how SGLT2 inhibitors affected inflammation in HF. This approach infers causal relationships between exposures and outcomes by using genetic variants as instrumental variables. Genetic variants associated with the SLC5A2 gene and HbA1c levels were chosen from the Genotype-Tissue Expression (GTEx) project and the eQTLGen consortium. In their study, Guo et al. (2024) used two datasets of 14,824 and 575,531 people of European ancestry, respectively, to obtain genome-wide association study (GWAS) data on 92 inflammatory biomarkers. A meta-analysis of 26 cohorts yielded the GWAS data for HF, which included 930,014 controls and 47,309 HF cases. Leukemia inhibitory factors and CXCL10 (C-X-C motif chemokine 10), which is present in chromosome 4 and one of the 92 inflammatory biomarkers, were linked to SGLT2 inhibition and heart failure. According to MR analysis, 17.85% of the effect of SGLT2 inhibition on HF was mediated by CXCL10, the main inflammatory cytokine linked to HF. This study identifies CXCL10 as a potential therapeutic target for HF and offers genetic evidence in favor of the anti-inflammatory effects of SGLT2 inhibitors. Since this is an MR study, the global heterogeneity test in the inverse variance-weighted (IVW) was conducted using Cochrane’s Q statistic to assess heterogeneity among the genetic instruments. They computed the F-statistic for the chosen SNPs to address weak instrument bias in the instrumental variable analysis. Neither horizontal pleiotropy nor any indication of heterogeneity among the genetic tools was found (Guo et al., 2024).

Luo et al. (2024) conducted a two-sample, two-step Mendelian Randomization (MR) analysis to determine the circulating proteins mediating the causal effects of SGLT2 inhibition on HF. The study evaluated the following: 1) the causal relationship between SGLT2 inhibition and HF; 2) its impact on 4,907 circulating proteins; and 3) the correlation between HF and proteins driven by SGLT2 inhibition. Previous studies were used to derive genetic variants associated with SGLT2 inhibition. The Heart Failure Molecular Epidemiology for Therapeutic Targets (HERMES) consortium provided the genetic links for HF, while the deCODE study provided the protein data. Leucine-rich repeat transmembrane protein 2 (LRRTM2) emerged as a key protein associated with both SGLT2 inhibition and HF, and the results showed that SGLT2 inhibition decreased the risk of HF. Through its interaction with Neurexin-1 (NRXN1), LRRTM2 contributes to the formation of synapses (de Wit et al., 2009; Yamagata et al., 2018). It has been discovered that the proteins NRXN1, NRXN3, and NCAM2 are produced by nerve cells in every region of the heart (Wang et al., 2022). Idiopathic dilated cardiomyopathy is linked to mutations in the NRXN1 gene, indicating that LRRTM2 may affect heart disease by controlling NRXN1 (Sajid et al., 2017). According to co-localization analysis, mediation analysis revealed that LRRTM2 was responsible for 24.6% of the effect of SGLT2 inhibition on HF. This study identifies LRRTM2 as a putative mediator and demonstrates a causal link between SGLT2 inhibition and HF. To evaluate the stability of the MR results, they used the heterogeneity and horizontal pleiotropy tests. The MR-PRESSO method and MR Egger regression were done which did not reveal any significant horizontal pleiotropy, and there was no discernible variation in the estimation of the effect of SGLT2 inhibition on HF in IVW and MR Egger analyses (Luo et al., 2024).

## 4 Discussion

To the best of our knowledge, this is the first systematic review that examines the genomic impact of SGLT2 inhibitors in heart failure. The findings collectively highlight the multifaceted impact of SGLT2 inhibitors on HF, emphasizing how they affect gene expression, circulating protein profiles, and inflammation modulation.

The main findings of our systematic review are:

• SGLT-2i treatment modifies the gene expression profile in EAT, reducing inflammation, immune cell infiltration, and markers of programmed cell death like ferroptosis. These may also help lower oxidative stress in EAT of SGLT-2i-treated subjects. A reduction in ferroptosis in EAT might be another way that SGLT-2i lowers its pro-inflammatory state, which in turn improves myocardial function. However, further research is required to substantiate this theory.
• Genetically predicted SGLT2 inhibition reduces HF risk, mediated significantly by the inflammatory biomarker CXCL10.
• It is identified that LRRTM2 is a critical mediator of SGLT2 inhibitors’ impact. LRRTM2 levels are genetically linked to reduced HF risk. Co-localization analysis confirms shared genetic loci between LRRTM2 and HF.

SGLT2 inhibitors have been shown to significantly reduce the expression of CXCL10, a chemokine involved in the pathophysiology of heart failure. This reduction may help mitigate inflammation and immune cell infiltration, which are key processes closely linked to the advancement of heart failure. CXCL10 has been shown to influence cardiac hypertrophy and fibrosis resulting from pressure overload, along with the functional impairments that characterize heart failure. Notably, the beneficial effects of SGLT2 inhibitors on heart failure can be attributed to the modulation of CXCL10, making this pathway a promising therapeutic target. To enhance the cardiovascular benefits of SGLT2 inhibitors, future treatments could explore strategies that inhibit CXCL10.

Another potential therapeutic approach is the modulation of LRRTM2 to improve cardiac resilience, particularly for patients with genetic predispositions to heart failure. Gene-editing techniques or targeted protein therapies could be explored to maximize the cardioprotective benefits of LRRTM2. While these findings are promising, large-scale clinical trials are essential to validate these mechanisms and translate them into actionable treatments.

The purpose of this systematic review was to evaluate how SGLT2 inhibitors affect human genomic markers. The species, heart failure models, and SGLT2 inhibitor dosages used in animal studies varied greatly, making it difficult to compare the results directly to clinical data from humans. Numerous studies on animals looked at molecular pathways without validating their applicability to patients with heart failure. Despite exclusion, animal model findings remain critical in forming hypotheses about SGLT2 inhibitors’ genomic effects.

Wu et al. (2013) highlighted the genetic component of SGLT2 inhibition in HF by exploring its effects using SGLT2-global-knockout (KO) mice. Despite the absence of SGLT2, dapagliflozin continued to exert cardioprotective effects, suggesting that its mechanisms might be independent of the SGLT2 protein itself. Single-cell RNA sequencing reveals that dapagliflozin significantly alters gene expression profiles in macrophages and fibroblasts, particularly those involved in inflammation and fibrosis pathways. The drug’s ability to modulate these genetic expressions underscores macrophage-mediated pathways as pivotal targets, rather than SGLT2, in mitigating HF progression (Wu et al., 2023).

Another study by Chen et al. (2022) uncovers the genetic mechanisms underlying the cardioprotective effects of dapagliflozin (DAPA) in a normoglycemic rabbit model of chronic heart failure (CHF). The results show that DAPA ameliorates cardiac fibrosis by downregulating the TGF-β1/Smad signaling pathway, which is a major regulator of the expression of fibrotic genes. Genes involved in extracellular matrix deposition, which are essential for the development of fibrosis, are influenced by this pathway. DAPA identifies a genetic mechanism independent of glucose regulation by modulating gene expression patterns and inhibiting TGF-β1/Smad signaling, which reduces fibrosis and improves myocardial structure and function (Chen et al., 2022).

### 4.1 Sodium-glucose cotransporters and SGLT2 inhibitors

The movement of glucose and sodium ions across cell membranes depends on a family of transmembrane proteins called sodium-glucose cotransporters (SGLTs). The tissues where SGLTs are frequently expressed include the small intestine, renal tubules, and the heart.

**FIGURE 3:**
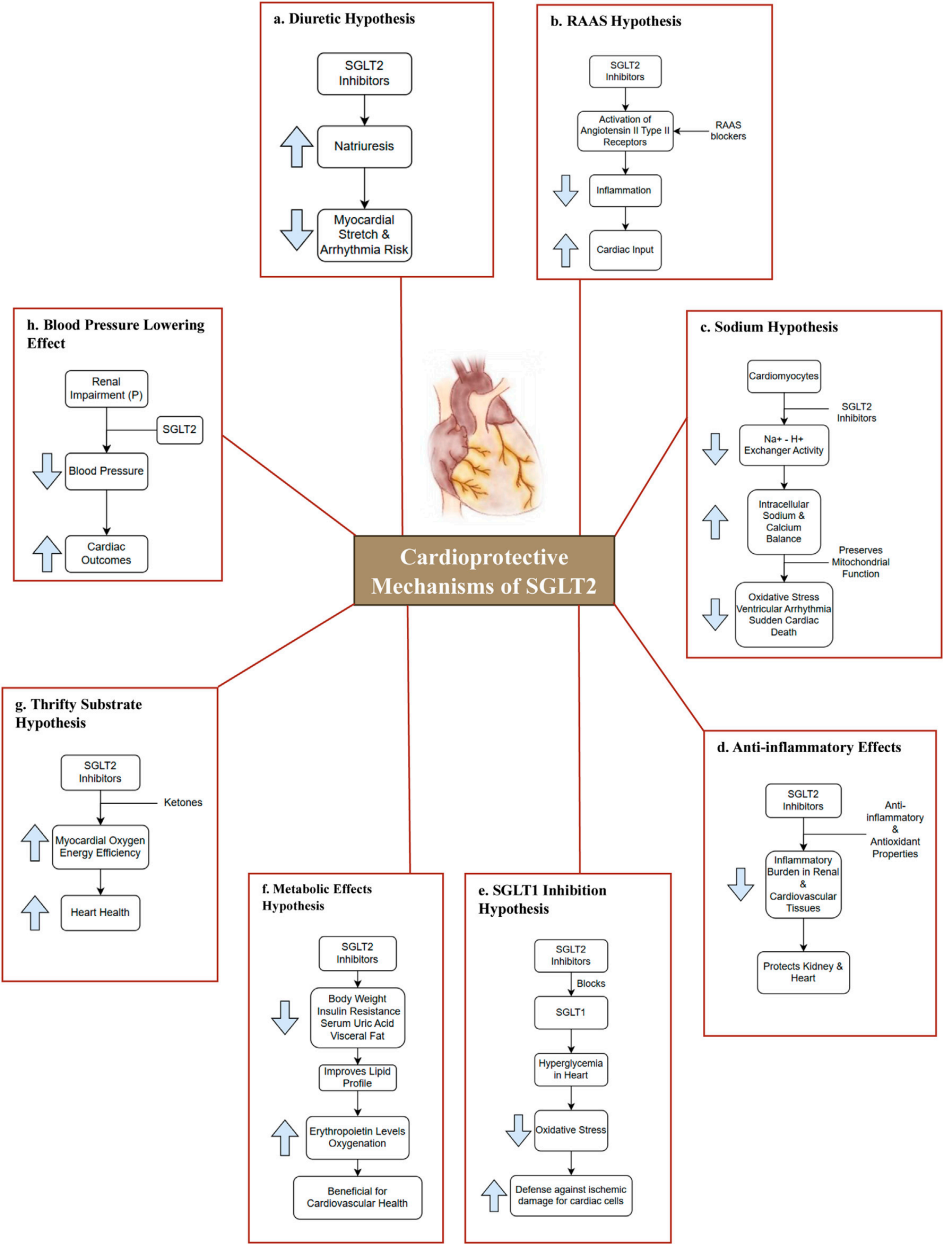
Cardioprotective mechanisms of SGLT2. **(a)** Diuretic Hypothesis: SGLT2 inhibitors lower blood pressure via osmotic diuresis and reduced arterial stiffness, decreasing afterload and enhancing cardiovascular outcomes (Ferrannini et al., 2017; Bertero et al., 2018; Lytvyn et al., 2017); **(b)** RAAS Hypothesis: SGLT2 inhibitors may activate angiotensin II type II receptors, promoting vasodilation and enhancing cardiac output (Muskiet et al., 2015); **(c)** Sodium Hypothesis: SGLT2 inhibitors regulate Na^+^-H^+^ exchange, preserving mitochondrial function and reducing oxidative stress, protecting against arrhythmias and sudden cardiac death (Bertero et al., 2018; Bell and Yellon, 2018); **(d)** Anti-inflammatory Effects: SGLT2 inhibitors exert anti-inflammatory and antioxidant effects, reducing inflammation in renal and cardiovascular tissues, thereby protecting the heart and kidneys (Ye et al., 2017; Ojima et al., 2015); **(e)** SGLT1 Inhibition Hypothesis: Certain SGLT2 inhibitors also inhibit SGLT1, lowering hyperglycemia-induced oxidative stress and enhancing cardiac protection against ischemic injury (Bell and Yellon, 2018; Van Steenbergen et al., 2017); **(f)** Metabolic Effects Hypothesis: These drugs reduce body weight, insulin resistance, uric acid, and visceral fat, improves lipid profiles, and increases erythropoietin, enhancing oxygenation and reducing inflammation for better cardiovascular health. (Ribola et al., 2017); **(g)** Thrifty Substrate Hypothesis: SGLT2 inhibitors boost ketone production, enhancing cardiac energy use, oxygen efficiency, and providing antioxidant and antiarrhythmic benefits. (Al Jobori et al., 2017); **(h)** Blood Pressure Lowering Effect: SGLT2 inhibitors lower blood pressure through diuresis and reduced arterial stiffness. This decrease in both systolic and diastolic pressures improves cardiovascular outcomes, even in renal impairment (Chilton et al., 2015). Abbreviation: P-Patients; RAAS-renin-angiotensin-aldosterone system.

The glucose transporters encoded by SLC2A1 (solute carrier family 2 member 1) (SLC2A1 solute carrier family 2, 2024), SLC5A (solute carrier family 5 member 1) (SLC5A1 solute carrier family 5, 2024), and SLC50A (Solute Carrier Family 50 Member 1) (SLC50A1 solute carrier family 50, 2024) belong to three families. In humans, SLC5A encodes SGLTs. The 12 genes that makeup SLC5A have been identified in various tissues, and each one has a distinct distribution and function (Youssef et al., 2023). Several hypotheses have been proposed to explain the cardioprotective effects of SGLT2 inhibitors in Figure 3 (Ferrannini et al., 2017; Bertero et al., 2018; Lytvyn et al., 2017; Muskiet et al., 2015; Bell and Yellon, 2018; Ye et al., 2017; Ojima et al., 2015; Van Steenbergen et al., 2017; Ribola et al., 2017; Al Jobori et al., 2017; Chilton et al., 2015).

These potential cardioprotective mechanisms are still not fully understood at the genetic level. Though it has significant cardio-reno protective actions, it is limited by an increased occurrence of urinary tract and vaginal infections, along with euglycemic diabetic ketoacidosis (Tentolouris et al., 2019).

## 5 Limitations

The number of studies is very few, and only two of these three studies had a large sample size. Secondly, the analysis is retrospective, which may compromise the accuracy and applicability of the results. While preclinical models provided valuable insights, more direct human studies are needed to validate these findings. Variations in study methodologies may introduce bias and limit direct comparisons. Among the included studies, two were Mendelian randomized and one was a cross-sectional clinical trial. The methods used to assess gene expression varied significantly, which may affect the comparability of results. The 2 MR studies used an existing dataset, and the other study used the tissue of patients with HF, which may contribute to discrepancies. It is crucial to understand that MR studies investigate association and not direct causation. While the reviewed MR studies indicate that CXCL10 inhibition and LRRTM2 modulation are genetically linked to SGLT2 inhibitor benefits in HF, these findings must be validated through direct experimental models.

Despite significant findings, the exact genetic pathways mediating the effects of SGLT2 inhibitors remain incompletely understood. Ongoing research is actively exploring these clinical observations’ genomic underpinnings to further refine our understanding of the context-specific factors that may influence individual patient responses.

## 6 Strength and future direction

The large sample sizes in these studies enhance the generalizability of the findings, strengthening the biological plausibility of the observed associations. These studies contribute significantly to the growing body of evidence regarding the cardioprotective effects of SGLT2 inhibitors beyond glycaemic control. While these strengths add credibility to the studies, the methodological differences between them make it challenging to synthesize the results. Future research should aim for standardized procedures and replication across diverse populations to confirm these findings.

## 7 Conclusion

This systematic review highlights the genomic insights into the impact of SGLT2 inhibitors on heart failure. The evidence underscores the multifaceted benefits of these inhibitors, particularly their ability to modulate inflammation, gene expression, and circulating protein profiles. Key findings include the downregulation of proinflammatory genes, the identification of CXCL10 as a critical inflammatory biomarker mediating therapeutic effects, and the role of LRRTM2 in reducing heart failure risk. These findings emphasize the potential of SGLT2 inhibitors as pivotal components of heart failure management, offering a pathway toward personalized medicine. From a genomic perspective, recent studies have provided insights into the genetic factors that may influence the response to SGLT2 inhibitors identifying genetic biomarkers and pharmacogenomic profiles associated with SGLT2 inhibitor efficacy could inform personalized treatment approaches and optimize therapeutic outcomes for patients with heart failure. It may channel us to a new therapeutic frontier!

## Data Availability

The raw data supporting the conclusions of this article will be made available by the authors, without undue reservation.
