# Supplementary material for: Genomic insights about the effect of sodium-glucose cotransporter 2 inhibitors: a systematic review
Source: Front Genet. 2025 May 30;16:1571032. doi: 10.3389/fgene.2025.1571032 (PMC12162637; doi:10.3389/fgene.2025.1571032)
Supplement: Supplementary file 1 [file DataSheet1.pdf]

The Risk of Bias in Non-randomized Studies of Interventions (ROBINS-I)

Study	D1		D2	D3	D4		D5	D6	D7
	A	B			A	B			
Kasperova BJ et al. (2024)	Low	Low	Low	Low	Low	Low	Low	Low	Low
Wenqin Guo et al. (2024)	Moderate	Serious	Low	Low	Low	Low	Low	Low	Low
Jinlan Luo et al. (2024)	Moderate	Serious	Low	Low	Low	Low	Low	Low	Low

D1: Bias due to confounding

D2: Bias in classification of interventions

D3: Bias in selection of participants into the study (or into the analysis)

D4: Bias due to deviations from intended interventions

D5: Bias due to missing data

D6: Bias in measurement of the outcome

D7: Bias in selection of the reported result
